# Conference Reports: WORKSHOP ON INTELLIGENT PROCESSING FOR PRIMARY METALS Gaithersburg, MD August 29–30, 1989

**DOI:** 10.6028/jres.095.046

**Published:** 1990

**Authors:** James G. Early

**Affiliations:** Scientific Advisor to the Director, Materials Science and Engineering Laboratory, National Institute of Standards and Technology, Gaithersburg, MD 20899

The Materials Science and Engineering Laboratory (MSEL) of the National Institute of Standards and Technology (NIST), the Office of Industrial Programs of the Department of Energy (DOE), and the American Iron and Steel Institute (AISI) co-sponsored a Workshop on Intelligent Processing for Primary Metals held at NIST on August 29–30, 1989. Attendance was by invitation and the more than 80 participants were primarily senior technical staff and managers from steel, aluminum, and copper companies.

Although the United States is a leading contributor to the world’s materials science base, it is beginning to lag in the cost-effective implementation of this knowledge. U.S. industry does not enjoy leadership in the introduction of new materials technologies into consumer products, as it does in defense systems. Other nations have initiated government-industry programs aimed at the development of advanced processing technology. Advanced manufacturing techniques increasingly demand materials of greater reliability and uniformity at competitive cost. Specific demands for properties and performance can be met by control of the processing of the material from synthesis or raw material production to forming/finishing.

Such process control, or “Intelligent Processing of Materials” (IPM), is based on four elements: a process model that relates specific materials properties at each stage of the processing to the final properties; sensors (measurement technology) which can measure the appropriate materials properties in real-time during processing; materials property data which must be coupled with the real-time measurements in the process model; and rapid computational capability for incorporation of sensor data, process model evaluation, and process variable control into an integrated, automated control strategy. The IPM concept differs greatly from conventional automated materials processing. In conventional practice, only the process variables (temperature, pressure, etc.) are automatically controlled to pre-set values which, nevertheless, still allow the microstructure and properties of the material to deviate significantly from the desired values. A critical feature of intelligent processing is the shift from off-line measurements on finished products to on-line measurements in real-time to control processing.

The past 5 years have witnessed extensive advances in the enabling technologies needed for intelligent processing of materials. These include: advanced sensors for on-line monitoring of material and process parameters, the knowledge base and materials characterization techniques to develop process models relating process parameters to material properties, and hierarchical computer control strategies for implementing artificial intelligence/expert systems concepts. Several recent national programs have been initiated to integrate elements of these advances to control the processing of advanced materials such as gallium arsenide crystals, powder metals, and composites. Similar opportunities now appear to exist in the primary metals industries.

The goal of this industry-led workshop was to highlight the recent advances in sensing, modeling, and process control, to identify areas of need in the primary metals industries, and to develop a strategy for implementation of research results. Industry, university, and government participants assessed information provided by researchers and operating staff from industry to develop a research agenda for coupling the advancing state of materials processing in the primary metals industries.

## 1. History of the Workshop

The genesis of this workshop may be traced to two events. First, the signing into law in late 1988 of the Steel and Aluminum Energy Conservation and Technology Competitiveness Act that authorizes DOE and NIST to carry out coordinated programs in support of the primary metals industries, primarily steel, aluminum, and copper. NIST would concentrate on providing instrumentation and measurement R&D. The second event was the January 1989 forum at Northwestern University to identify long-range research opportunities for the North American steel industry. The industry participants concluded that research opportunities could best be addressed in the context of three specific long-range development projects: direct production of liquid steel, near-net shape casting, and finishing and coating operations.

To capitalize on these events, planning was initiated through a steering committee, chaired by Lyle H. Schwartz, Director, MSEL/NIST, with representatives from steel, aluminum, and copper trade associations, individual companies, the academic community, and Federal agencies. The consensus was to attempt to address the process control needs of the broad spectrum of primary metal industries and the role of intelligent processing concepts in solving these needs by focusing on three generic areas: primary metal production/refining, production of near-net shape products, and finishing/coating to final properties. Emphasis would be on future, advanced processes and technologies.

## 2. The Workshop

The Organizing Committee, chaired by James G. Early, MSEL/NIST, was pleased to have participation by representatives of the aluminum and copper industries because the workshop structure is applicable to the processing of these metals. An overlap or commonality of process control needs is likely in some areas of processing. The success of this workshop could lead to other industries using this approach to develop process control priorities. Although the strategy was to achieve a workshop relevant to steel, aluminum and copper, the program contained a strong orientation toward steel-related issues. Since early in this decade, the steel industry has worked to develop a consensus on technical advances needed to improve traditional production practices and on identifying the future steel-making technologies. Thus, the steel industry was particularly well-positioned to play an important role and make major contributions to the workshop resulting in the strong emphasis on steel.

The core of the workshop was organized into three working sessions:

- Direct Liquid Metal Production
- Near-Net Shape Casting
- Finishing and Coating

Within these areas, the key elements of intelligent processing were stressed. The relationships between fundamentals and processing were explored through the integration of process modeling, sensor technology, and control strategies. Leading off the first day of the Workshop was an introduction to intelligent processing of materials concepts through applications to aerospace and other advanced materials followed by an overview of the process control research needs for the production of steel and aluminum. The three working sessions, responsible for Direct Liquid Metal Production, Near-Net Shape Casting, and Finishing/Coating, took place in the afternoon of the first day and the morning of the second day to permit a wide range of inputs from the participants. Coordinated presentations were given in these working sessions on the status of sensors, process models, and control approaches, the available technology, and the benefits to relevant research. After the morning sessions on the second day, a brief, verbal synopsis of the deliberations in each of the three working sessions was presented to the assembled workshop participants.

Between the first and second days working sessions, Deputy Secretary of Commerce Thomas J. Murrin, spoke to the Workshop on “New Metals Technologies: Making the Government-Industry Connection Work.” In his remarks, Mr. Murrin discussed the three key issues facing industry as it develops new metals technologies: the need to improve the quality of products and services; the need to take advantage of new technologies being developed overseas; and the need for continued and expanded government-industry-labor cooperation. While the metals industry’s health will be determined primarily by its own efforts, there are appropriate areas in which the Federal Government can contribute. Deputy Secretary Murrin reported on a number of policy initiatives underway related to taxes and tax credits, antitrust laws, and a broad review of technology-innovation policies and programs to determine if other changes are needed.

## 3. Plenary Session

The first session was devoted to a series of invited presentations to prepare the participants in the three working sessions with the necessary background and global view needed to achieve the Workshop goal. The first speaker, W. Barker (DARPA) described the concept of intelligent processing of materials and reviewed the national programs supported by DARPA. In a companion talk, D. Backman (General Electric Company) reviewed the application of IPM concepts to advanced aerospace materials. The industry perspective was presented in the final two talks: I. Hughes (Inland Steel) “Long Range Research -North American Steel’s Competitive Edge”; and R. Bonewitz (Alcoa) “Sensor, Process Models, and Control Needs in the Aluminum Industry.”

## 4. Direct Liquid Metal Production Session

Co-Chairmen:

A. W. Cramb
Carnegie-Mellon University
J. Kor
Timken Company

The session was organized into two parts: the first part was an information exchange between experts on sensor technology, process control, modeling, and the application of advanced computer decision making techniques, while the second part was to define and prioritize specific needs in the area of intelligent processing of liquid metals. Within each area specific needs were identified. Speakers included: J. Kor (Timken Company); P. Koros (LTV Steel Company); J. Fay (ASARCO, Inc.); A. McLean (University of Toronto); D. Hardesty (Sandia National Laboratory); C. Alcock (Notre Dame University); Y. Kim (Lehigh University); R. Guthrie (McGill University); M. Shah (IBM); and S. Ray (NIST).

The sensor area was considered to be the area of highest importance and it was the consensus of the group that sensor development and implementation should be the major focus of any endeavor. Three separate groups of sensors were distinguished: continuous temperature sensors; continuous chemical sensors for liquid metal and hot, dirty gases; and, physical sensors to measure reaction intensity. The area of process modeling was identified as the second most important area for research at this time. Two separate groups of needs were outlined: Process Control Models; and In-Detail Process Models. In the area of process control there was a concern that current “Artificial Intelligence” techniques might not be applicable to on-line control in a broad sense; however, in certain well-defined circumstances it may be useful. In addition, it was felt that the computer science involved in process control and decision making was sufficiently rapid that a mechanism should be set up so that appropriate advances can be implemented within the industry in a reasonable time frame. Three separate groups of needs were identified: Operator Feedback and Instruction; System Integration; and Technology Transfer. A copper industry participant reported that many of the sensors discussed would also be of use to the copper industry and further identified the sensors that overlapped those needed by the steel industry.

## 5. Near-Net Shape Casting Session

Co-Chairmen:

W. E. Eckhart, Jr.
U.S. Department of Energy
R. Sussman
ARMCO, Inc.

This session was conducted in two parts. The first day was characterized by presentations made by experts in their respective fields. Each presentation generated a modest number of questions within the subject area. It is noteworthy that no process-specific problems were detailed during the first-day presentations, to the disappointment of some of the participants. The second day of this session was started by brief presentations made by individuals having hands-on experience with near-net shape casting of metal. Despite the obvious differences between the various casting methods, needs were categorized into three distinct areas: liquid metal handling, casting, and strip collection. Speakers included: M. R. Moore (USS Division of USX); R. A. Gleixner (Battelle Memorial Laboratories); L. T. Shiang (Inland Steel Company); Y. Sahai (Ohio State University); H. N. G. Wadley (University of Virginia); and J. A. Walton (ARMCO, Inc.).

Eight sensors were identified as critical to the successful development of a commercial strip casting process: continuous temperature measurement of the meet; continuous temperature of the substrate surface; rapid on-line strip thickness sensing; on-line hot strip inspection system; continuous topographic sensing of substrate; liquid metal level sensor; liquid metal inclusion sensor; and liquid metal nitrogen sensor. It was recognized that each of these must be employed in a manner in which it could be actively used to control the outcome of the process, and not merely in a passive role to provide information. Attempts were made to determine the range and precision needed for each of the sensors. In many cases, the identified range was quite accurate, but the estimated precision reflected that required of the product; the sensor may require greater sensitivity. In the area of mathematical modeling, it was agreed that most models are process-specific. Nonetheless, there exist many commercially available models of the two principal phenomena involved in near-net shape casting, heat transfer and fluid flow. It was determined after considerable discussion that the industry would benefit from a comprehensive review of such models currently available, along with their respective strengths and weaknesses, and a strategy for implementation of selected models. In the area of process control, it was recognized that the overall state of knowledge of near-net shape casting makes it difficult to design a comprehensive control system at this stage of development. It was determined that it would be meritorious to undertake an intelligent process simulation project to serve as a model for the metals industry. Shortly after the close of the workshop, a participant from the copper industry submitted an analysis that summarized similarities and differences in the sensor/model/control needs for copper and steel near-net shape casting.

## 6. Finishing and Coating Session

Co-Chairmen:

D. Watanapongse
Inland Steel
A. Van Clark, Jr.
NIST

The session consisted of two meetings designed to facilitate interaction between national experts and industry experts. The goal of the first meeting was to understand and assess the states of knowledge in intelligent processing; namely, product/process knowledge, process modeling, sensors, integrated process control, and artificial intelligence. The product/process relationships for steel substrates and coated steel were presented along with the needs for future improvements. Subsequent presentations were in sensor development, process modeling, and advanced control. The goal of the second meeting was to address the issues and resolutions that can enhance the success of future research programs to develop and apply intelligent processing technologies. Speakers included: D. Reinbold (Bethlehem Steel); P. Southwick (Inland Steel); S. Denner (National Steel); K. Brimacombe (University of British Columbia); J. Monchalin (IMRI Canada); A. V. Clark, Jr. (NIST); L. Lowry (Jet Propulsion Laboratory); and A. Meystel (Drexel University).

Although there is a wide range of sensor needs in the finishing and coating processes, five sensors were identified as having highest priority: continuous temperature measurement of strip; on-line measurement of chemical composition and phase identification in coatings; measurement of surface topography and surface chemistry of strip; measurement of lubricant film thickness; and measurement of mechanical properties/microstructure. Further attempts to prioritize among the five or to define specific ranges and accuracy of the sensors were considered inappropriate by the group because sensor requirements must be specified as an integral part of process modeling and control system development. Attempts were made, however, to estimate the developmental time for the sensors based on current understanding of available principles and technologies. In the coating processes, much work is needed to understand and model coating adherence mechanisms for a range of coating materials, steel substrates, surface morphology, and processing parameters. In addition, press performance of coated materials in terms of stampability and powdering characteristics must be studied and modeled. Engineered surfaces and microstructural engineering were cited as the important areas needing further modeling studies. Since intelligent processing involves implementation and also a wide range of multidisciplinary backgrounds, the significance of project team formation, technology transfer, designed-in safety and maintenance were discussed at length. Intelligent processing has definitive roles in the finishing and coating processes, in terms of new product and process design and on-line process control. Specific recommendations are: Develop real-time expert models for control to supplement process models; and develop implementable intelligent processing systems from hot rolling to coating, with emphasis on continuous processing.

## 7. Summary

The research agenda developed at this industry-led workshop is summarized in NIST Special Publication 772, “Intelligent Processing for Primary Metals.” According to the participants, successful development and implementation of advanced processing concepts for the production of steel, from raw steel through finished steel products, will be enhanced through the formation of broad-based, multidisciplinary teams focusing on generalized approaches and solutions to well-defined tasks. Specific recommendations for critically-needed sensors, process models, and control strategies are reported for the three working sessions.

For information on collaborative IPM research opportunities at NIST or a copy of the workshop report, NIST Special Publication 772, send a self-addressed mailing label to Dr. James Early, Materials Building, Room B309, NIST, Gaithersburg, MD 20899.

